# Unlocking the potential of engineered microbes in immunotoxin-based cancer therapy

**DOI:** 10.3389/fmicb.2025.1603671

**Published:** 2025-06-05

**Authors:** Quan Wang, Rui Cao, Yuxing Xie, Zhuoyi Zhang, Xianguo Li, Yan Zhang, Haolin Luo, Hui Yao, Ping Xue, Shuai Ni

**Affiliations:** ^1^School of Pharmacy, Xianning Medical College, Hubei University of Science and Technology, Xianning, China; ^2^Guangdong Provincial Key Laboratory for Plant Epigenetics, College of Life Sciences and Oceanography, Shenzhen University, Shenzhen, China; ^3^Department of Anesthesiology, People’s Hospital of Xinzhou, Wuhan, China; ^4^Department of Anesthesiology, Yunxi People’s Hospital, Shiyan, China; ^5^Department of Neurosurgery, Union Hospital, Huazhong University of Science and Technology, Wuhan, China

**Keywords:** immunotoxin, cancer therapy, engineered microbes, circuits, tumor penetration

## Abstract

Immunotoxins (ITs), as targeted cancer therapies, confront limitations including off-target effects, immunogenicity, and inadequate tumor penetration, hindering clinical translation. Advances in tumor microenvironment (TME) understanding and genetic engineering have enabled engineered microorganisms such as attenuated *Salmonella*, *E. coli* Nissle 1917, and modified eukaryotic platforms (e.g., yeast, microalgae) to colonize tumors and act as efficient hosts for IT production. By integrating ITs into these microbes and employing precise circuits (e.g., phage lysis systems, signal peptide fusions), controlled secretion of recombinant immunotoxins (RITs) can be achieved. Balanced-lethal systems further enhance plasmid stability for sustained therapeutic delivery. This review highlights strategies leveraging engineered microbes to amplify IT efficacy, exemplified by preclinical successes like *Salmonella*-delivered TGFα-PE38 and *E. coli*-expressed anti-PD-L1-PE38. However, challenges persist, including dynamic TME interactions, systemic infection risks, manufacturing complexities and regulatory uncertainties demand resolution. By synergizing microbial targeting with RIT, this approach offers transformative potential for cancer therapy, yet requires multidisciplinary innovation to address technical, safety, and regulatory barriers for clinical adoption.

## 1 Introduction

**Figure 1:**
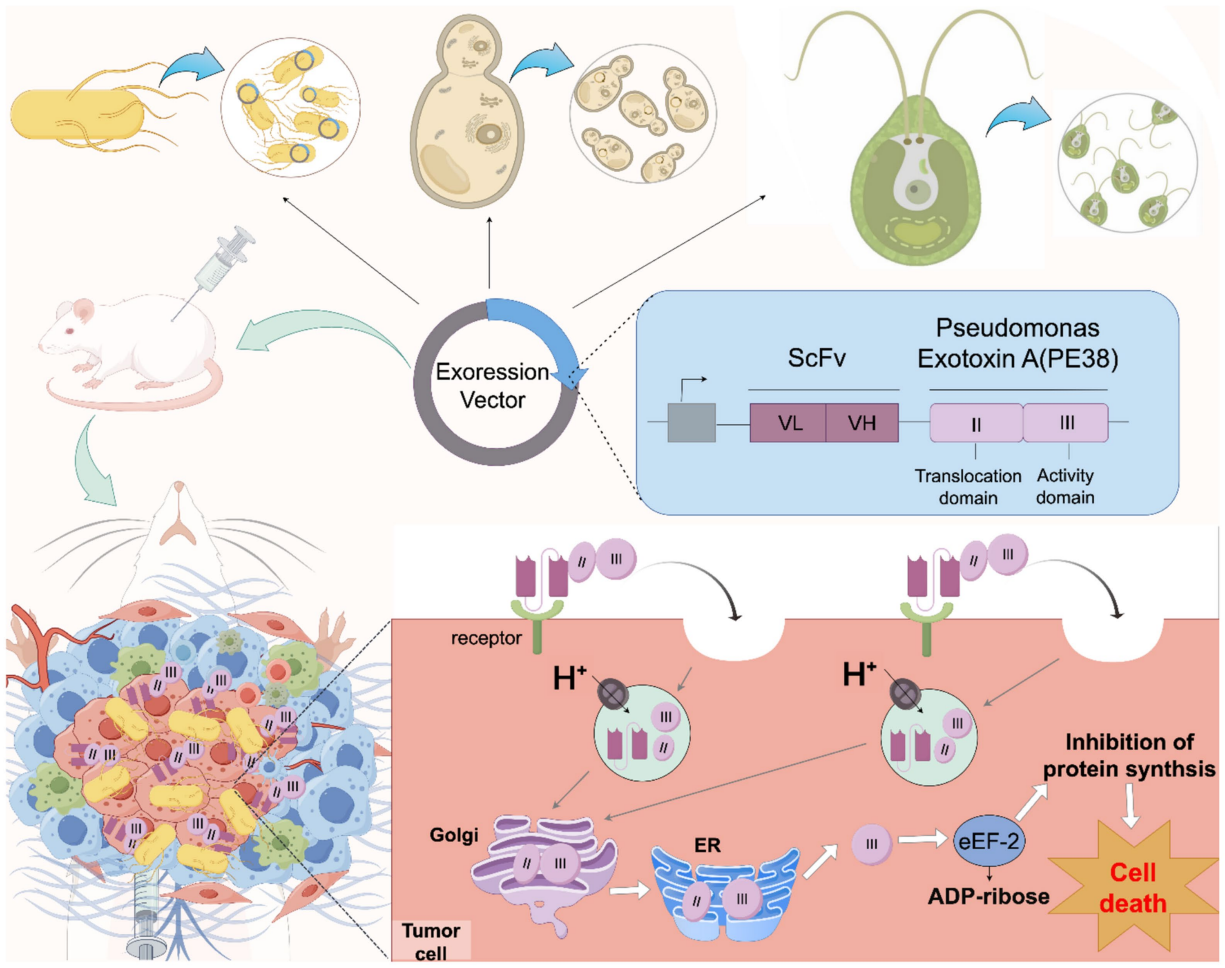
Schematic diagram of engineered microbes promoting IT cancer therapy. Engineered microorganisms (including bacteria and eukaryotes) with cancer colonization or targeting capabilities can serve as hosts for expressing RITs. Loading RITs into these microorganisms, combined with the design of specific release circuits, enables the microorganisms to release ITs within tumor tissues. Once released, the IT first binds to the target cell surface receptor in a targeted and cell-specific manner, then is internalized into the cell through receptor-mediated endocytosis, and directed to endosomes. Subsequently, in the endosomal lumen acidified by an ATPase proton pump, the toxic moiety is cleaved by an endosomal acidic pH-activated protease called furin. Taking Pseudomonas Exotixin A as an example, the cleaved PE38 active (catalytic) domain is transported to the trans-Golgi network and then the endoplasmic reticulum (ER) via retrograde trafficking before being released into the cytosol. Finally, the PE38’s active (catalytic) domain in the cytosol exerts its effector function by inactivating eukaryotic elongation factor 2 (eEF2) through catalyzing adenosine diphosphate (ADP) ribosylation, which inhibits protein synthesis and ultimately leads to cell death, thus achieving the killing of cancer cells.

Cancer remains a global threat, with nearly 20 million new cases and about 10 mil-lion deaths yearly (Bray et al., 2024). Despite various anticancer methods and drugs, minimizing damage to normal cells while maximizing cancer cell killing remains a constant pursuit (Anand et al., 2023; Feo et al., 2022; Katz et al., 2022; Labanieh and Mackall, 2023). Targeted therapy, which precisely identifies and targets cancer cell features while sparing normal tissues, has garnered significant attention (Lee et al., 2018). ITs and antibody-drug conjugates (ADCs) are both effective targeted therapy agents with similar structures. They share almost the same targeting components but differ in their cytotoxic payloads and conjugation methods. Targeting components, responsible for locating cancer-specific antigens, are usually composed of monoclonal antibodies or antibody fragments. ITs also used ligands binding to specific receptors, such as cytokines, chemokine receptor ligands and growth factors as targeting units (Babavalian et al., 2019; Janthur et al., 2012; Kreitman, 2006; Spiess et al., 2017). The cytotoxic payloads of ITs are typically protein toxins or their modified derivatives from bacteria (e.g., *Pseudomonas aeruginosa* exotoxin A, diphtheria toxin, or anthrax toxin), plants (e.g., ricin, saporin, or gelonin), humans (e.g., proapoptotic proteins and RNA enzymes), or other sources (e.g., chelona toxin) (Bachran and Leppla, 2016; Gill et al., 2024; Knödler and Buyel, 2021; Lu et al., 2021; Shafiee et al., 2019). In contrast, ADCs have a broader range of cytotoxic payloads, including microtubule inhibitors, DNA-damaging agents, RNA inhibitors, immunomodulators, proteasome inhibitors, small molecules, multi-drugs, phosphate prodrugs, and proteolysis-targeting chimeras (PROTACs) (Chen et al., 2020; Phuna et al., 2024; Tsuchikama et al., 2024; Wang et al., 2024; Xi M. et al., 2024). Due to the nature of their cytotoxic payloads, ADCs usually rely on chemical conjugation. ITs, have more flexible conjugation methods, as they can be either chemically conjugated or directly expressed as fusion proteins via amino acid linkers (Bacauanu et al., 2023; Khoshbakht et al., 2024; Oghalaie et al., 2024; You et al., 2021). Despite their similarities, ITs and ADCs have had different outcomes. Over 15 ADCs have been approved for clinical use, with hundreds more in clinical trials. In contrast, only a few ITs have been approved (Colombo et al., 2024; Khirehgesh et al., 2021; Kim et al., 2020). The main reason is that some key issues in IT design and development remain unresolved. These include off-target effects, where normal cells expressing the target are attacked, leading to systemic toxicity; immunogenicity caused by heterologous toxins and antibody molecules; the inability of IT molecules to efficiently permeate solid tumors to reach effective therapeutic concentrations; and the lack of efficient cytoplasmic delivery pathways after internalization (Balkhi et al., 2025; Dhillon, 2018; Markides et al., 2025). Recent studies have shown that some microorganisms can colonize cancers and tend to proliferate in the hypoxic and immunosuppressive TME, significantly influencing tumor progression (Kwon et al., 2024; Xu et al., 2018; Yu et al., 2020). Through bioengineering, these microbes can be utilized for cancer therapy in various ways, such as specifically infecting tumor tissue, activating innate and adaptive immunity, releasing toxins to kill cancer cells, competing with cancer cells for nutrients to impede tumor growth, or carrying therapeutic agents to treat cancer (Copland et al., 2024; Moon et al., 2020; Zhang et al., 2024). This presents a great opportunity for ITs, which allows directly expressed and processed in engineered hosts through recombinant gene construction (Figure 1). Many historical limitations of IT based cancer therapy can now be overcome using these microbial hosts. Here, we comprehensively review the key considerations for using microorganisms to express and deliver ITs for tumor treatment, as well as current research progress. We look forward to strengthening our cancer-fighting arsenal and expanding IT-based therapeutic strategies.

## 2 Immunotoxin therapy for cancer: progress and issues

Although conjugation methods are not restricted, most immunotoxins currently depend on recombinant expression frameworks for direct production across various hosts (Zuppone et al., 2019). This approach is favored over chemical conjugation as it is more likely to yield a uniform product. Consequently, immunotoxins are now commonly designated as RITs. Since initial development by Thorpe in 1978, RITs have evolved through four iterations, resulting in four distinct generations of products (Thorpe et al., 1978). Researchers have endeavored to enhance this selective cancer-cell killing agent by modifying all of its structures, including the target units, payloads, and conjugation methods (Golichenari et al., 2025; Obozina et al., 2025; Wang et al., 2025; Wang et al., 2024). For instance, in the third and fourth-generation RITs, murine antibodies have been supplanted by humanized or fully human antibodies to reduce immunogenicity (Hauser et al., 2023; Waldmann, 2019). Furthermore, complete antibodies have been replaced with smaller fragments such as single chain antibody (scFv), and even nanobodies (VHH) to improve tumor penetration (Morgan et al., 2023; Naemi et al., 2023; Wang et al., 2024; Xi X. et al., 2024). Payload optimization has also been pursued, such as toxin structure optimization and epitope deletion (Golichenari et al., 2025; Hu et al., 2016; Mazor et al., 2015). Moreover, internalization efficiency, escape speed from vesicles to the cytoplasm, *in vivo* half-life, and administration routes are all optimized (Wang et al., 2025; Wei et al., 2018). However, these advancements do not always bring benefits and are sometimes accompanied by challenges. Using human toxin payloads can lower immunogenicity, but in vivo activity is often hindered by endogenous inhibitors. For example, Granzyme B (GrB) is inhibited by serine protease inhibitor B9, which greatly weakens its killing effectiveness (Hlongwane et al., 2018). Additionally, compared to plant and bacterial toxins, human toxins frequently lack a translocation domain, making them more susceptible to lysosomal degradation rather than migrating to the cytoplasm for therapeutic effect after cell internalization (Mungra et al., 2019). In summary, despite various improvement efforts, the clinical application prospects of RITs are still worrying. Some optimization measures beyond RITs themselves may solve this situation, such as the engineered microorganisms with unique abilities that we are currently focusing on.

## 3 Engineered microbes: potential vehicles for enhancing RIT cancer therapy

Research on the tumor microbiome has unveiled a complex ecosystem comprising tumor cells and intracellular microbes. This complexity is reflected in both the diversity of indigenous microbes, which includes various bacteria (e.g., B*acteroides*, *Enterococcus*, *Faecalibacterium*, *Ruminococcus*, *Clostridium*, *Lactobacillus*, and *Actinomyces*), fungi (e.g., *Yeast*, *Candida*, *Blastomyces*, and *Malassezia*), and multiple viruses, and the intricate interrelationships within the system (Dohlman et al., 2022; Luca et al., 2021; Nejman et al., 2020; Sepich-Poore et al., 2021). On one hand, these microbes shield tumors by influencing their occurrence, development, metastasis, heterogeneity, and immune evasion. On the other hand, they compete with cancer cells for nutrients, activate innate and adaptive immunity to kill cancer cells, produce toxins to damage cancer cells, and modulate the tumor microenvironment to enhance treatment efficacy (Galeano Niño et al., 2022). These “double-edged” microbes, when genetically engineered, could maximize benefits and minimize drawbacks, holding great potential for future cancer treatment. This is why microbe-based cancer therapy (MCT) is gaining increasing attention recently (Zheng and Chen, 2024).

MCTs are actually not novel. Over 100 years ago, therapies using inactivated *Streptococcus* and *Serratia marcescens* (Coley’s toxins) injected into malignant tissues were employed and resulted in sarcoma regression (Coley, 1991). Another example is *Bacillus Calmette-Guérin* (BCG), a live attenuated strain of bovis *Mycobacterium tuberculosis* variant initially developed as a tuberculosis vaccine. It has been approved by the FDA for the treatment of bladder cancer (Boorjian et al., 2021). Unlike past methods using natural microbes or their toxins, employing microbes to create antitumor vaccines or deliver therapeutic agents shows much greater promise (Shende and Basarkar, 2019). Many recent studies substantiate this viewpoint. For example, using attenuated *Salmonella typhimurium* (SAM-FC) to deliver ClyA and FlaB significantly suppresses metastases and primary tumors, and VNP20009 to deliver Sgc8c (nucleic acid aptamer) targeting PTK7 in pancreatic cancer shows good effect, engineering *Listeria monocytogenes* secretes phospholipase (plcA, plcB) and hemolysin LLO to deliver tumor-specific antigen TAAs to alter TME and increase immune killing (Hassan et al., 2019; Nguyen et al., 2024; Xiao et al., 2024). Many cancer therapies and drugs, such as immune checkpoint blockades, antibodies, ADCs, and chemotherapy drugs, have an upper limit on their therapeutic effects and scope of application (Schuster et al., 2021). For example, the clinical benefit rate of immune checkpoint blockades is typically below 30% (Kalbasi and Ribas, 2020). However, combining these agents with cancer-colonizing microbes and genetic engineering techniques shows a high probability of breaking through such limitations. This also applies to RITs. In fact, attempts to use engineered microbes to express and deliver RITs have already begun.

A few pioneering research projects with ingenious design have already achieved promising initial results and are expected to yield broader applications in the near future (Table 1). For example, the engineered *Salmonella typhimurium* ΔppGpp strain can express and deliver the RIT composed of TGF*α* (transforming growth factor alpha, a ligand targeting epidermal growth factor receptor) and PE38 (*Pseudomonas* exotoxin A fragment), which can significantly inhibit mouse solid tumor growth (Lim et al., 2017). The RIT constructed from the non-neutralizing anti-TNF-α antibody Pigbak#2 and PE38, produced and delivered by *E. coli* BL21(DE3) Δlpp, have shown strong antitumor activity in mouse melanoma models (Hu et al., 2022). The construction of the anti-PD-L1 (programmed cell death ligand 1) antibodies and PE38 expression system in the *Nissle 1917* strain has demonstrated superior suppression effects in mouse subcutaneous tumor models via intravenous injection (Li et al., 2025). Although “hitchhiking therapy” has shown promising results in mouse models, its potential risks, particularly in clinical settings, must be closely watched (Tang et al., 2022). Researchers have implemented many sophisticated regulatory circuit designs to avoid risks, enhance therapeutic effects, and increase controllability. These designs are worth highlighting and promoting. First, host selection is crucial. Many microbes preferentially infect tumor tissue, but only those that are naturally safe or engineered to be attenuated can be used to minimize infection and dissemination risks. For example, the *Salmonella* ΔppGpp strain with *relA* and *spoT* gene mutations is deficient in guanosine 5′-diphosphate-3′-diphosphate synthesis. This strain has almost lost its ability to invade mammalian cells and has good safety (Liu et al., 2022). The *E. coli* Nissle 1917 strain is not only sensitive to the immune system, does not produce pathogenic enterotoxins or cytotoxins, but can also antagonize pathogenic *E. coli* and has a good safety record for *in vivo* applications (Yu et al., 2020). To ensure the host can release RITs, advanced regulatory circuits have been introduced. Lim et al. successfully delivered immunotoxins using a Salmonella phage lysis system (pLYS) with three Salmonella phage genes. They also enabled efficient RIT secretion by fusing a soil cellulose-degrading bacterium’s cellulase (Psp) signal peptide to the RIT’s N terminus. Similarly, Li et al. fused yebF to the N terminus of αPD-L1-PE38 for secretion. In contrast, Hu et al. knocked out Braun’s lipoprotein-encoding gene to engineer a leaky strain that continuously releases Pigbak#2-PE38 extracellularly. Moreover, RIT recombinant genes need stable maintenance in the host without loss in the absence of antibiotic pressure. Lim et al. achieved this using a balanced-lethal host-vector system, which mutates the essential glmS gene and introduces a recombinant GlmS+ plasmid to ensure every surviving host carries the RIT and GlmS+ plasmid. In contrast, Hu et al. demonstrated that incorporating kanamycin resistance and a ColE1 origin into the recombinant plasmid ensures its stability for 8 days without antibiotic pressure. Finally, some other designs are also effective. For example, Li et al. demonstrated that extending the linker sequence between anti-PD-L1 and PE38 can reduce steric hindrance and enhance binding affinity. The use of inducible promoters can enhance controllable secretion, and adding the KEDL sequence to the RIT expression frame is believed to promote immunotoxin retention in the cytoplasm and boost toxin efficacy (Hu et al., 2022; Jeong et al., 2014; Li et al., 2025; Lim et al., 2017).

**Table 1:** Immunotoxins loaded by engineered microbes.

Immunotoxin	Engineering host	Special circuits	Tumor type and administration route	Reference
TGF*α*-PE38	*S. typhimurium* △ppGpp	(a) glmS based balanced-lethal system: a hosts with *glmS* gene deficiency rely on a recombinant *GlmS+* plasmid for survival, ensuring plasmid stability in vivo. (b) pLYS plasmid lysis system: composed of three genes from the Salmonella phage (iEPS5), effectively lyses the host and releases RIT. (c) Psp secretion system: a novel cellulase 32AA signal peptide from a cellulose-degrading bacterium *Paenibacillus* sp. *EC003* allows effective release of RIT.	Colon cancer, Breast cancer & Tail vein injection	Lim et al. (2017)
Pigbak#2-PE38	*E. coli* BL21(DE3)△lpp	(a) “leaky” system: knock out Braun’s lipoprotein gene, construct engineered strains with outer membrane integrity deficiency to promote the release of cellular contents. (b) “Trojan Horse” tactic: Through engineered hosts, on the one hand induce TNF-α overexpression, on the other hand release TNF-α -targeting immunotoxins. Via TNF-α, both TNF-α receptors and immunotoxins form a sandwich, thereby promoting RIT internalization and killing cancer cells.	Melanoma tumor & Intratumoral injection	Hu et al. (2022)
αPD-L1-PE38	*E. coli* Nissle 1917	(a) yebF^SP^ system: the signal peptide of YebF protein is involved and ensures effective RIT secretion. (b) Antibody and toxin linker optimization: Linkers KASGG, (G4S)2, (G4S)3, A3(G4S)1, A3(G4S)2, and A3(G4S)3 have been tested to find the optimal one that reduces steric hindrance between antibodies and toxins.	Colon cancer & Intratumoral and intravenous injection	Li et al. (2025)

In summary, engineering microbes to express and deliver RITs for cancer therapy shows promise, especially with bacterial hosts. Engineered bacteria have successfully targeted and released RITs in solid tumors, inducing cancer cell apoptosis and showing good therapeutic effects (Shuwen et al., 2024; Tieu et al., 2024). However, RITs sometimes require post-translational modifications that bacteria cannot perform. Eukaryotic hosts can provide these modifications for fully functional RITs, yet there are no studies on using eukaryotic vehicles for RIT delivery in cancer treatment, despite their presence in tumor tissue (Zuppone et al., 2019). A recent study successfully engineered a yeast strain to express and secrete PD-1 high-affinity microantibodies. Oral administration targeted and alleviated cancer in a mouse intestinal tumor model (Rebeck et al., 2025). Additionally, a PDA-CV@PD-1 inhibitor delivery system, using microalgae coated with chemicals and loaded with immune checkpoint inhibitors, demonstrated the potential of microalgae to deliver drugs to tumors (Zeng et al., 2025). Both yeast and microalgae are promising eukaryotic platforms for expressing recombinant proteins like RITs, offering post-translational modifications for full functionality and potentially safer *in vivo* applications compared to bacterial hosts. Thus, engineered yeast and microalgae could become important vehicles for delivering RITs in cancer therapy.

## 4 Perspectives and challenges of IT-loaded microbes

In oncology, microbes have transitioned from being mere suppliers of essential anti-tumoral agents, including antibiotics such as doxorubicin and bleomycin, enzymes like L-asparaginase and arginine deaminase, and toxins such as Coley toxin and diphtheria toxin, to being recognized as live therapeutic entities. Their innate ability to target and proliferate within tumors significantly boosts their value in cancer therapy. This is because they cannot only activate immune responses against cancer cells but also serve as precise vehicles for delivering therapeutic agents to the TME. As of now, over 50 live microbial agents for treating various malignancies have completed clinical trials, and this number continues to grow (Nguyen et al., 2024). Immunotoxins, due to their facile incorporation into microorganisms, are anticipated to considerably augment their anticancer efficacy and broaden their applications through these “living agents.”

Despite its great prospects and several successful cases, the clinical application of live microbial agents loaded with IT faces the following major challenges: Firstly, in terms of the accuracy and controllability of drug delivery, although many studies have shown that microbes can proliferate rapidly in the TME and many MCTs have ultimately achieved significant intratumoral colonization effects in vivo, the main pathways and mechanisms by which bacteria reach tumors are still unclear. Further research on the main pathways and mechanisms of live microbes reaching tumors is of great significance for the clinical translation of IT-loaded microbes. In addition, due to the dynamic nature of the TME, when these IT-carrying microbes function in the tumor, both the TME and the tumor itself may change. This may expand bacterial colonies into normal tissues, thereby causing systemic infection. The dynamic TME also poses a huge challenge for precisely controlling IT release. This is because when tumor tissue declines due to treatment, “live microbial agents” and the IT cargo they produce may instead increase. More circuit design or additional antibiotic control is needed to balance this inconsistency. Moreover, this kind of live drug will apparently not follow the conventional pharmacokinetic characteristics, which also poses challenges to clinical drug monitoring and use. Secondly, regarding safety and patient individual differences, despite using attenuated or non-pathogenic microbes, the bacteria can still cause infection or excessive immune activation, posing safety risks. A patient’s immune status significantly impacts the effectiveness of live biotherapeutic products. Those with strong immune systems may quickly eliminate therapeutic bacteria, reducing treatment efficacy. Conversely, immunocompromised patients face higher infection risks and require careful management during treatment. Additionally, human microbiota varies between individuals, which can affect the performance of live biotherapeutic products. For example, a patient’s gut microbiota may interact with therapeutic bacteria, changing their growth, metabolism, and pharmacological effects, potentially leading to unstable treatment outcomes. Thirdly, in terms of production processes and quality control, unlike conventional drugs, the production process of these live microbial agents cannot rely on filtration or heat sterilization to eliminate other pathogenic bacteria, posing new challenges for production and quality control. Although additional resistance genes and antibiotics can be introduced during production to control other pathogenic bacteria, this approach carries the risk of resistance gene transfer within the body, potentially leading to antibiotic resistance. Finally, there is a lack of authoritative or official regulatory documents specifically targeting these live microbial agents.

## 5 Conclusion

In conclusion, engineered microbes present a promising and innovative approach to enhancing immunotoxin-based cancer therapy. Their unique capabilities to target and proliferate within tumors offer significant advantages, addressing several limitations of traditional immunotoxins. However, the clinical application of these live microbial agents faces substantial challenges, including ensuring the accuracy and controllability of drug delivery, managing safety concerns and patient individual differences, overcoming complexities in production processes and quality control, and navigating the lack of specific regulatory guidelines. Future research needs to focus on optimizing microbial delivery systems, improving our understanding of tumor-microbe interactions, and establishing appropriate regulatory frameworks. Despite these hurdles, the potential of engineered microbes to revolutionize cancer treatment and improve patient outcomes remains substantial, warranting continued exploration and development in this exciting field.
